# The development of a milk-derived nanovesicle with its potentials for nucleic acid delivery and bioconjugation

**DOI:** 10.7150/ntno.113165

**Published:** 2025-08-22

**Authors:** Bhanubhong Prommalee, Chalermchai Pilapong

**Affiliations:** Laboratory of BioMolecular Imaging, Molecular and Cellular Biology, Department of Radiologic Technology, Faculty of Associated Medical Sciences, Chiang Mai University, Chiang Mai, Thailand.

**Keywords:** Nanovesicle, Nucleic acid, Drug delivery, Bioresource

## Abstract

To date, nanotechnology facilitating cellular penetration toward specific target sites has widely been adopted in several studies. Some use chemical synthetic approaches for enhancing drug delivery whereas some attempt to select bioresources. Biomaterial substances that are thought to be less toxic to cells are currently in demand since they have been proven to demonstrate drug delivering capability. We thus explored the production of a nanovesicle from bioresource that is supposed to be one of the potential carriers facilitating target delivery. Herein, a breast milk-derived substance was used as a bioresource for producing the milk-derived nanovesicle. The results showed that a small, spherical and negatively charged nanovesicle was successfully prepared by using an extrusion method without the use of any chemical substances and solvents. The nanovesicle showed satisfactory profiles in terms of cytotoxicity without any sustained secretion of cytokines. The nanovesicle also provided delivering potential towards different nucleic acids including single strand DNA, microRNA, and mRNA. Moreover, the nanovesicle was also shown to have bioconjugation capability. These outcomes illustrate the benefits of using breast milk as one of the most vigorous biomaterial substances that can be adopted into a nanovesicle-based drug delivery strategy.

## Introduction

Over many decades various nucleic acid delivering technologies have been developed, especially non-viral delivery of target genes into the cells, i.e., lipid nanoparticles, polymers, micelles, etc. These can be the ways of dealing with some limitation of adopting viral vectors in nucleic acid delivering including low immunogenicity, high biocompatibility, and biodegradability 1. Lipid nanoparticles (LNPs) have been shown especially a capable of well-established entrapment and delivery for nucleic acids such as siRNA, mRNA, miRNA, ASOs, and DNA 2. LNPs have been used since their main components comply by having the properties of increasing drug stability, as providing drug-degraded protection from nucleases. Because of their capable of promoting target-drug delivery, furthermore, they have been proposed to reinforce personalized medicine 3-4. One of the famous LNPs-based nanocarriers is liposome, which is delineated by amphiphilic lipid bilayers containing aqueous compartments in spheric vesicular forms. Its functionality is applicable of facilitating delivery of nucleic acids since its lipid bilayer performs greater biocompatibility toward cellular compartment upon simply being fused by cell membrane 5. Liposomes are characterized as having amphiphilic lipid bilayers containing aqueous compartments in spherical vesicular forms. Its functionality is applicable for facilitating the delivery of nucleic acids since its lipid bilayer performs greater biocompatibility toward cellular compartments when simply fused to a cell membrane 5. A Liposome can be generated by chemical reagents and natural resources. Previously, biological phospholipids, i.e., monosialoganglioside, 1,2-distearoryl-sn-glycero-3-phosphatidyl choline, egg phosphatidylcholines, and sphingomyelin were thought to be the chemicals involved in the formulation of liposome. Additional compositions such as polyethylene glycol (PEG) are used to enhance drug circulation time 6. Targeting ligand anchored nanovesicles were also developed in order to maintain site-specific drug delivery and release 4. Cationic liposomes are used to form complexes with siRNA and have less cytotoxic properties. Liposomes made of sodium cholate combined with 1,2-Dioleoyl-3-trimethylammonium propane (DOTAP), 1,2-dioleoyl-sn-glycero-3-phospho-ethanolamine (DOPE), and C6 ceramide can form a complex with siRNA and are rapidly taken up by target cells 7.

Since natural substances are less toxic, biocompatible, and biodegradable, they have been considered beneficial as bioresource for nanodelivery preparation. Milk is promising bioresource that can be used for preparation of different nanodelivery platforms because of their physical and chemical properties, scalable, economical source as well as substantial nutrients 8-9. Nowadays, milk-derived delivery systems (MDDS) based on naturally assembled architectures produced from milk (milk fat globules, caseins, whey proteins, extracellular vesicles and exosomes) have been explored 10-11 and they have been utilized as natural drug carrier that can treat diseases and enhance human health. In addition, milk can be used to prepare different types of nanovesicles. Re-assembled casein micelles were shown to improve bioavailability of vitamin D with highly protective effect against gastric degradation 12. Heat-stable liposome can be also synthesized from milk fat globule membrane (MFGM) phospholipids without using any solvents 13. The obtained liposome was shown to increase the bioavailability of bioactive compounds 14.

Since there are benefits of milk-derived nanocarriers applicable to delivering bioactive compounds, especially, nucleic acids, we attempted to prepare a milk-derived nanovesicle without the use of any chemical reagents and sought to characterize its functionalities toward any application to drug delivery. We also provide evidence for useful applications of a chemically-modified approach which might be rewarding outcomes for bioconjugation associated with stipulated aspects of precision medicines.

## Materials and Methods

### Materials

Frozen human breast milk was kindly donated by Miss Siriprapa Jattukul. Dulbecco's modified Eagle's medium (DMEM) and Roswell Park Memorial Institute 1640 medium (RPMI1640) were purchased from Caisson Laboratories, USA. Fetal bovine serum (FBS), and Penicillin-streptomycin were purchased from Capricorn Scientific, Germany. LymphoprepTM was purchased from Serumwerk Bernburg, Geramany. 3-(4, 5-dimethylthiazolyl-2)-2, 5-diphenyltetrazolium bromide (MTT), 2, 7-dichlorofluorescein diacetate (H2DCFDA) and dimethyl sulfoxide (DMSO) were purchased from Sigma-Aldrich, USA). Fluorophore labeled single DNA strands were purchased from IDT (FAM-DNA1; /5' 6-Fluorescein/CCTGGCCCCTTGTAGGACACTTTGT, and Cy3-DNA2; /5' Cyanine3/AGAGTAGTCCACCAACAAAGCGTAT. Cyanine3 labeled single microRNA 34a strands (Cy3miR) were purchased from GenePharma, China. EZ Cap™ EGFP mRNA was purchased from APExBIO. Cyanine5-NHS ester (succinimidyl ester) was purchased from Thermo Scientific, USA. Fluorescein isothiocyanate (FITC) was purchased from Bio Basic Inc, Canada. BODIPY 581/591 C11 was purchased from Invitrogen, USA. Amicon® Ultra centrifugal filter was purchased from Millipore, Germany.

## Methods

### Nanovesicle formation

Human breast milk was suctioned and kept at -20 °C. In this process, the lipid solution was primarily disrupted by the 6 cycles of the freeze-thaw process. The solution was then segregated by bath sonication (37 kHz x 1 min) and a 3-time centrifuge (15,000 RPM x 15 min, 4 °C) process, respectively. The supernatants were collected in each round of centrifuge to be filled in the extruder with a holder/heating block (Avanti Research). In a typical extrude, 1 mL of the sample was loaded into one syringe connected to the extruder which contain a polyester filter support. The other syringe was also attached to the other side of the extruder. Placing the extruder on the heating block requires a temperature at 70 °C. To extrude the samples, we gently pushed the plunger of the syringe to transfer it to an alternate syringe 5 times (a total of 10 passes) through a 100-nm pore-sized polycarbonate membrane. A nanovesicle was then generated. The solutions were subsequently purified by using an Amicon® Ultra centrifugal filter (100K NMWCO) under centrifugation at 2800 g for 20 min at 25 °C. The purified nanovesicle were then kept at 4 °C for *in vitro* experiments, and lyophilized for Fourier transform infrared (FTIR) analysis.

### Nanovesicle characterizations

The particle size and zeta potential of the nanovesicles were measured using dynamic light scattering (DLS) with a Zetasizer Nano ZS (Malvern, Worcestershire, UK). Nanovesicle samples were diluted 10-fold prior to measurement, and the viscosity of the dispersant was set to that of water at 25 °C. The particle size and nanostructure of the nanovesicle were determined using a transmission electron microscope (TEM, Hitachi HT7800) with negative staining. The functional group of the nanovesicle (lyophilized) was analyzed using a FTIR spectrometer (BRUKER TENSOR27).

### Cell culture

Human epidermal keratinocyte (HaCaT) was maintained in Dulbecco's modified Eagle's medium (DMEM; Caisson Labs) with 10% fetal bovine serum (FBS), 1% Penicillin-streptomycin solution and was maintained at 37 °C under 5% CO_2_ atmosphere.

Peripheral blood mononuclear cells (PBMCs) were isolated from buffy coat obtained through a fresh blood donation from a local blood bank unit at Chiang Mai University Hospital, Thailand, by density gradient centrifugation using LymphoprepTM according to manufacturer's instructions. The isolated cells were maintained in a Roswell Park Memorial Institute (RPMI) 1640 medium with 10% fetal bovine serum, 1% Penicillin-streptomycin solution and were maintained at 37 °C under 5% CO_2_ atmosphere.

### Cell viability of the nanovesicle

HaCat cells at logarithmic growth phases were seeded at densities of 5.0x10^4^ cells into a 24-well cell culture plate for 24 h. After that, the cells were incubated with different amounts of nanovesicles for 24 h, and then were rinsed with PBS twice prior to adding MTT solution (5 mg/mL in PBS) at 500 µL/well for 4 h. After the incubation, the supernatant was aspirated, and 500 µL DMSO was then added to dissolve the precipitate. The absorbance value was measured at 570 nm with a UV-Visible spectrophotometer (Shimadzu, UV-2700i). The cell viability was relatively calculated as the percentage of untreated cells (control).

### ROS generation of the nanovesicle

A detection kit was used to evaluate the ROS generation. HaCaT cells at logarithmic growth phases were seeded at densities of 5.0x10^4^ cells into a 24-well cell culture plate for 24 h. After that, the cells were incubated with different amounts of nanovesicles for 24 h, and then were rinsed with PBS twice prior to incubating with 2, 7-dichlorofluorescein diacetate (DCFDA) for 30 min. After washing, the fluorescence intensity of DCF (green channel) within the cells was observed under fluorescence microscope (Nikon, Eclipse Ts2), and the signal intensity was quantified using Image J software.

### Measurement of secretory cytokines

For HaCaT, the cells at logarithmic growth phases were seeded at densities of 5.0x10^4^ cells into a 24-well cell culture plate for 24 h. After that, the cells were incubated with different amounts of nanovesicles for 24 h and 48 h. For PBMC, the cells (5.0x10^4^ cells) were incubated with different amounts of nanovesicles for 24 h and 48 h. After the desired time, the cell culture media were collected for determining secretory cytokines. TNF-alpha, IL-10, and IL-1beta levels were individually measured using a MILLIPLEX MAP Human Cytokine/Chemokine Magnetic Bead Panel immunology multiplex assay.

### Evaluation of nucleic acid delivery

The nanovesicle formation designed to directly encapsulate different types of nucleic acids was also completed and its capacity for being delivering into the cells was illustrated. In a typical fabrication of nano-delivery of nucleic acids, a single DNA strand and dual DNA strands (FAM-DNA1 and Cy3-DNA2), microRNA (Cy3-miRNA), and mRNA (EGFP-mRNA) were separately mixed with the lipid solution, following by the extruder and purification based on the method mentioned above.

To illustrate the capacity for the nano-delivery of nucleic acids into the cells, HaCat cells which had been seeded in 12-well culture plates were prepared prior to being treated with different amounts of the nano-deliveries for different lengths of time. Cellular accumulation of the nano-deliveries was performed with a fluorescent microscope and a flow cytometer (Beckman coulter, cytoFLEX).

### Functionality of bioconjugation

To exhibit functionality of bioconjugation by the nanovesicle, fluorescein isothiocyanate (FITC, Bio Basic Inc) and Cyanine5-NHS ester (succinimidyl ester) (Thermo Scientific) were used to covalently link to the membrane of the nanovesicle. In a typical protocol, the pure nanovesicle was incubated with FITC, Cy5-NHS for 1 h at room temperature, following by purification by Amicon® Ultra centrifugal filter (100K NMWCO). The FITC or Cy5 labeled nanovesicles were then incubated with HaCat cells. After desired time of incubation, the cellular accumulation of FITC or Cy5 labeled nanovesicles was observed using fluorescent microscopy and flow cytometry.

### Statistical analysis

Experiments were independently repeated, at least in triplicate. Data was presented as mean ± standard deviation (sd). Statistical tests were performed on Grapad Prism. Unpaired t-test was used to compare the means of two independent groups. One-way analysis of variance (ANOVA) was used to compare the means of three or more independent groups. Statistical significance was profound when the p-value was lower than 0.05.

## Results and Discussion

### Nanovesicle formation and characterization

**Figure 1:**
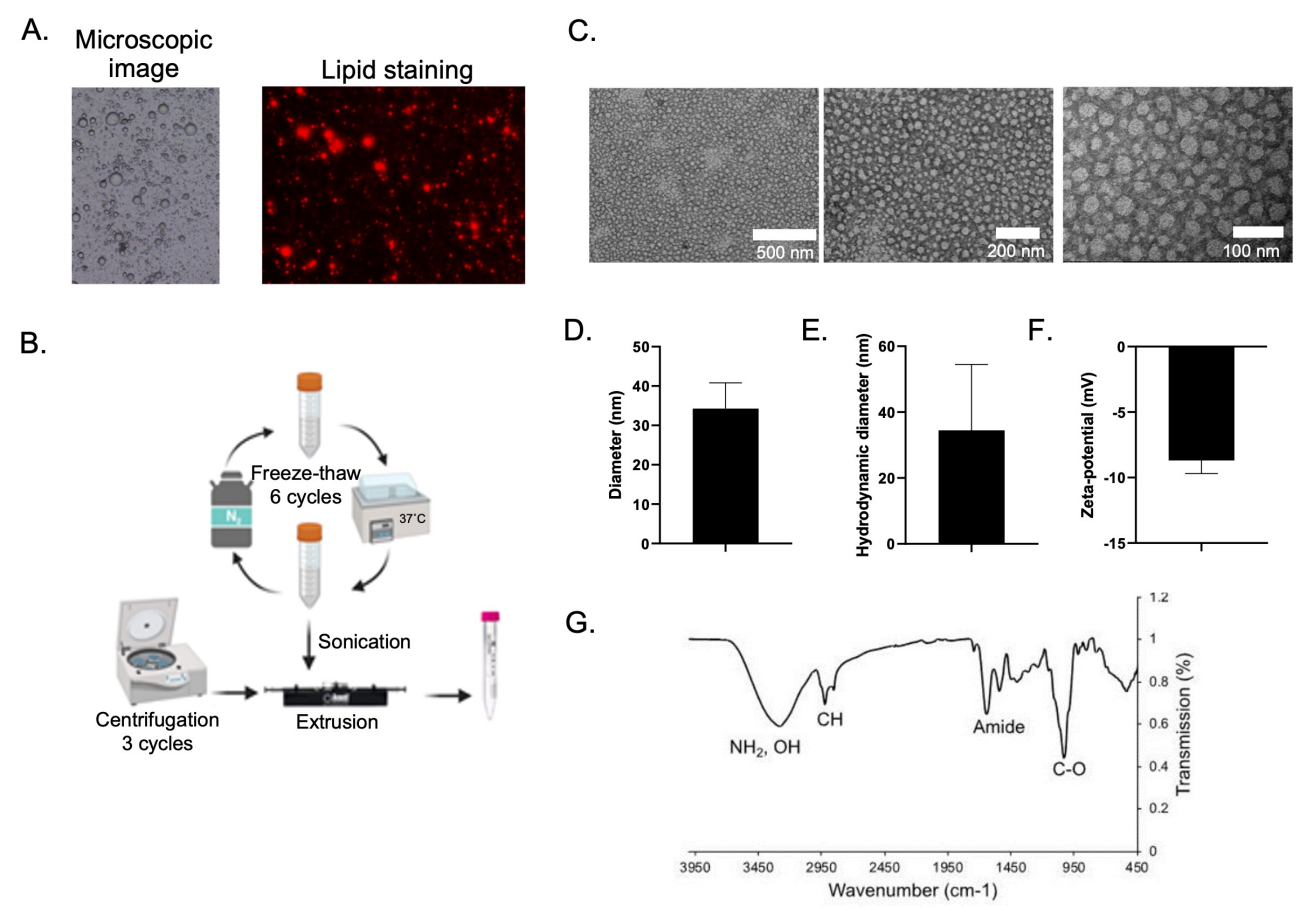
Nanovesicle formation and characterizations. (A) Microscopic image (left) and lipid staining image of breast milk suspension (right). (B) process of nanovesicle formation from breast milk. (C) of TEM images with different magnifications of the obtained nanovesicle. (D) physical size of nanovesicle measured from TEM images. (E and F) hydrodynamic diameter and zeta potential of the obtained nanovesicle, respectively. (G) FTIR spectrum of the obtained nanovesicle.

Our study revealed a process of nanovesicle formation derived from new biological substances like human breast milk. The process was aimed to illustrate the of use new materials which encourage biocompatibility and are less toxic for cells. In general, breast milk contains a high amount of lipids, especially in the form of MFG 15, generally consist of triglycerides surrounded by a structural membrane composed of phospholipids, cholesterol, proteins, and glycoproteins 16. Figure 1A, (left panel) showed MFG found in human breast milk used in this study. By staining the droplet with BODIPY 581/591 C11, a fluorescent probe for membrane lipid 17, fluorescent signal was observed at the membrane of the droplets (Figure 1A, right panel), suggesting that the membrane is made of a phospholipid. Furthermore, previous research found that the membrane of the lipid droplets is a 2-3 layered membrane composed of various phospholipids e.g., phosphatidylcholine (PC), phosphatidylethanolamine (PE), sphingomyelin (SM), phosphatidylserine (PS), and phosphatidylinositol (PI) 18. Therefore, breast milk can be used as a source for lipid based nanovesicle formation. Although MFGM in mammalian milk are compositionally similar but human MFGM contains more SM than others, resulting a better membrane stability 19,20.

The process of nanovesicle formation varies depending on the purpose of experiments and the availability of experimental resources. Sonication, which was one of methods used in our synthesis, was a crucial scenario since small unilamellar vesicles can be derived 21. Centrifugation applies the benefits of substance separation based on the weight of sedimentation 22 while a freeze-thaw process is a way of avoiding chemical hazards. We had developed these ideas for lipid segregating so that the lipids can be generated before constructing the nanovesicles by the extrusion technique. Figure 1B illustrates the nanovesicle formation process used for this study. In a typical manner, the milk suspension underwent a 6-cycle freeze-thaw-induced biodegradation. A process was then conducted to segregate phospholipid materials using sonication following by a 3-time centrifuge method. A process of extrusion had subsequently been adopted for constructing the nanovesicles. The assessments of the nanovesicles characteristics were then addressed in order to assure its physio-chemical properties. TEM analysis showed that the nanovesicles displayed small vesicles of spherical shape with a ~34 nm in diameter particle size (Figure 1C-D). The hydrodynamic diameter and zeta potential value were also determined to be ~34 nm and -8.7 mV, respectively (Figure 1E-F). Clearly, our biocompatible nanovesicle characteristics led to satisfactory profiles. Size is a considerable factor contributing to circulation half-life properties. In particular, size extended from 50 nm to 150 nm is necessary for drug delivery 23. Our nanovesicle's sizes and surface charges values were acceptable being in the suitable range according to an existing study 4.

### Nanovesicle toxicity and immune response

**Figure 2:**
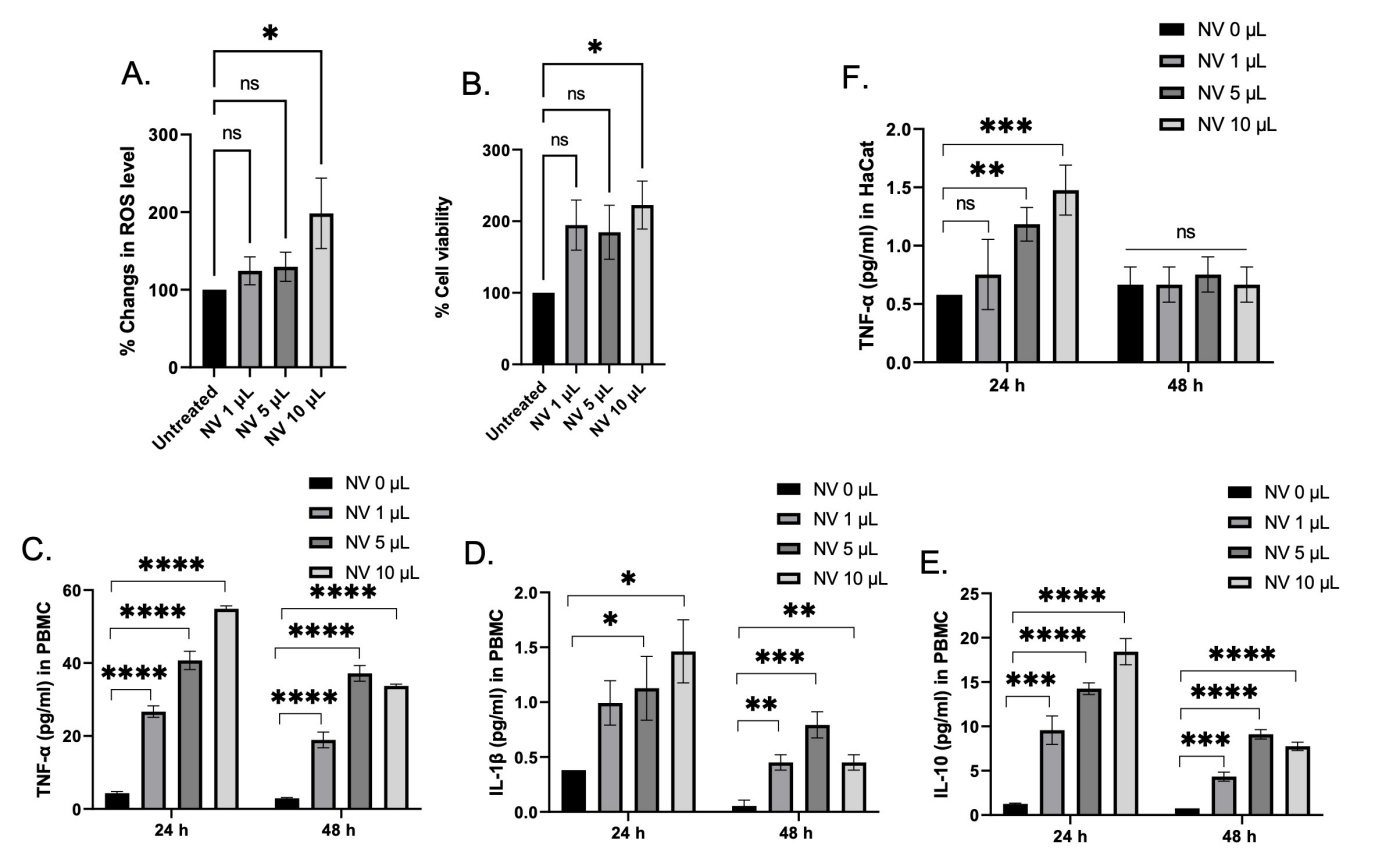
Nanovesicles Toxicity and Immune Response. (A and B) Change in ROS level and cell viability in HaCaT cells upon being treating with different amounts of the nanovesicles (NVs) for 24 h. (C-E) Quantification of different secretory cytokines including TNF-α, IL-1β, and IL-10 in PBMC after being treated with different amounts of nanovesicles at 24 h and 48 h. (D) Quantification of TNF-α level secreted from HaCaT cells after being treated with different amount of nanovesicles at 24 h and 48 h. ns = not significant; *p < 0.05, **p < 0.01, ***p < 0.001, ****p < 0.0001 by one-way ANOVA with Fisher's LSD test).

Despite the fact that the biocompatible substance used for nanovesicles construction was initially considered due to avoidance of the use of detergents toxicity yield from the anionic moieties, and particularly the hydrophilic parts is still noted. Therefore, nanovesicles' toxicity was evaluated. Firstly, HaCaT cells were used to study the effect of the nanovesicles on the promotion of intracellular ROS and cell viability. Despite the fact that ROS generation at 24 h had increased in a concentration manner compared with the untreated cells (Figure 2A), the nanovesicles seem to help conserve cellular survival since the viability profiles illustrate satisfactory outcomes. (Figure 2B), suggesting that breast milk-derived nanovesicles also did not demonstrate serious toxicity on the cells. To confirm that an immune response of the nanovesicles had occurred, PBMCs were chosen as the model for this investigation. Different secretory cytokines including TNFα, IL-10, and IL-1β levels were determined. At 24 h of incubation, the nanovesicles were found to induce PBMC to release TNFα, IL-10, and IL-1β in a concentration-dependent manner. It was not surprising that the nanovesicles were capable of inducing the release of the cytokines because of host immunity, inflammatory responses, and cell-mediated immune responses 24,25. Previously, some synthetic lipid nanoparticle was found to be potent activator of the inflammasome pathway, as indicated by robust release of IL-1β from PBMC. Compared with the synthetic lipid nanoparticles, our nanovesicles exhibited a much lower amount of secreted IL-1β, indicating that our nanovesicles was lower immunogenicity 26. For 48 h incubation, the amounts of secretory cytokines were found to decrease, comparing to a 24 h incubation. This suggests that the nanovesicles did not induce prolonged cytokine accumulation, and could not lead to promote cytokine storming. As for secretory cytokine in HaCaT cells, TNFα was selected to determine this because of its role in non-immune inflammatory response 27,28. It was found that the amount of TNFα were individually higher in a concentration-dependent manner among the groups comparing to the control at 24 h. In contrast, at 48 h of incubation, the levels of TNFα were found to decrease to the same levels of the control group (Figure 2E). Thus, the use of nanovesicles implemented in HaCaT cell lines was primarily not detrimental.

### Evaluation of nucleic acid delivery

**Figure 3:**
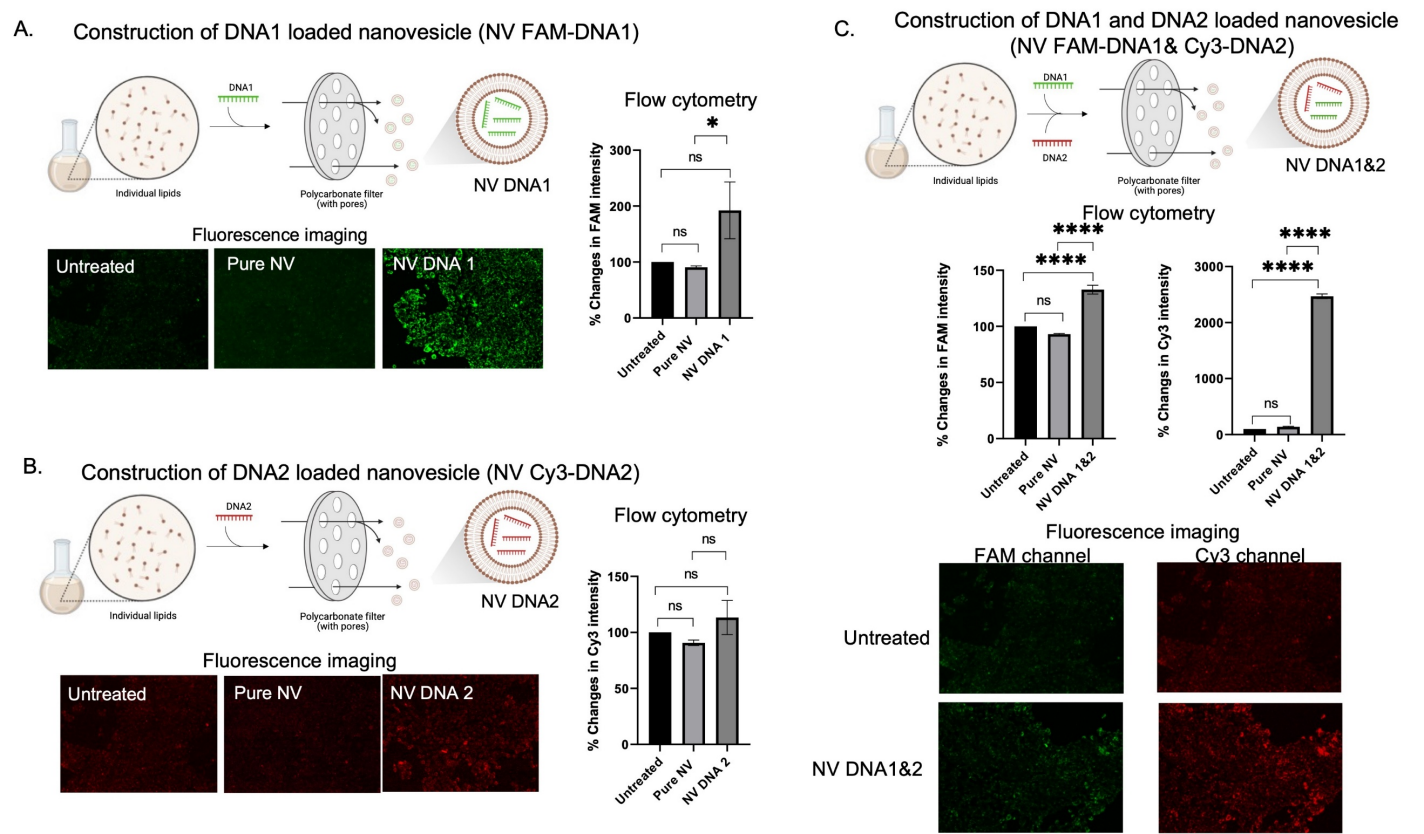
DNA encapsulations and delivery potentials of the nanovesicle. (A and B) Schematic illustration of the construction of single DNA (FAM-DNA1 or Cy3-DNA2) loaded nanovesicles along with their qualitative (fluorescence imaging) and quantitative (flow cytometry) analysis for intracellular accumulation. (C) Schematic illustration of the construction of dual DNA FAM-DNA1 and Cy3-DNA2) simultaneously loaded nanovesicles (along with their qualitative (fluorescence imaging) and quantitative (flow cytometry) analysis. (ns = not significant; *p < 0.05, **p < 0.01, ***p < 0.001, ****p < 0.0001 by one-way ANOVA with Fisher's LSD test).

Based on the merits of drug delivering being facilitated by nanovesicles, nucleic acid-like single strand DNA (ssDNA), microRNA, and mRNA was applied. For ssDNA delivery, DNA loaded nanovesicles were constructed using extruder sets. Two different DNA strands labeled with different fluorophores were used to evaluate the capability of in-cell drug delivery by nanovesicles (FAM for DNA1 and Cy3 for DNA2). When independently loading FAM-DNA1 and Cy3-DNA2 in nanovesicles (as shown in Figure 3A-B), greater signal intensity of DNA1 or DNA2 loaded nanovesicles (NV FAM-DNA1 or NV Cy3-DNA1) was observed, as compared to pure nanovesicle and the untreated group, suggesting that delivery of DNA1 or DNA2 by the nanovesicle revealed promising outcomes. Similarly, the quantification analysis by flow cytometer revealed that there was a significance increase in fluorescent signal in either DNA1 or DNA2 loaded nanovesicles. These results confirm the capability of nanovesicles to facilitate the delivery of single strand DNA. We then attempted evaluations of the simultaneous delivery of two different DNA strands (FAM-DNA1 and Cy3-DNA2) by the nanovesicles. As the results show in Figure 3C, quantitative analysis by flow cytometer revealed that DNA1 and DNA2 loaded nanovesicle (NV FAM-DNA1& Cy3-DNA2) displayed higher signal intensity of both FAM and Cy3 channels, compared to pure nanovesicle and untreated groups. In addition, the fluorescent imaging also illustrated that encouraging nanovesicle encapsulation toward dual DNA strands still displayed greater signal intensity with co-localization of the two different DNA strands (Figure 3C), suggesting that a single nanovesicle can successfully deliver multiple DNA strands into the cells. Encapsulation based on multiple DNA strands still illustrated higher intensity in the cells. It is possible that the effects of nanovesicle encapsulations highly dominate beyond other factors since the DNA strands used in the study are not too long.

**Figure 4:**
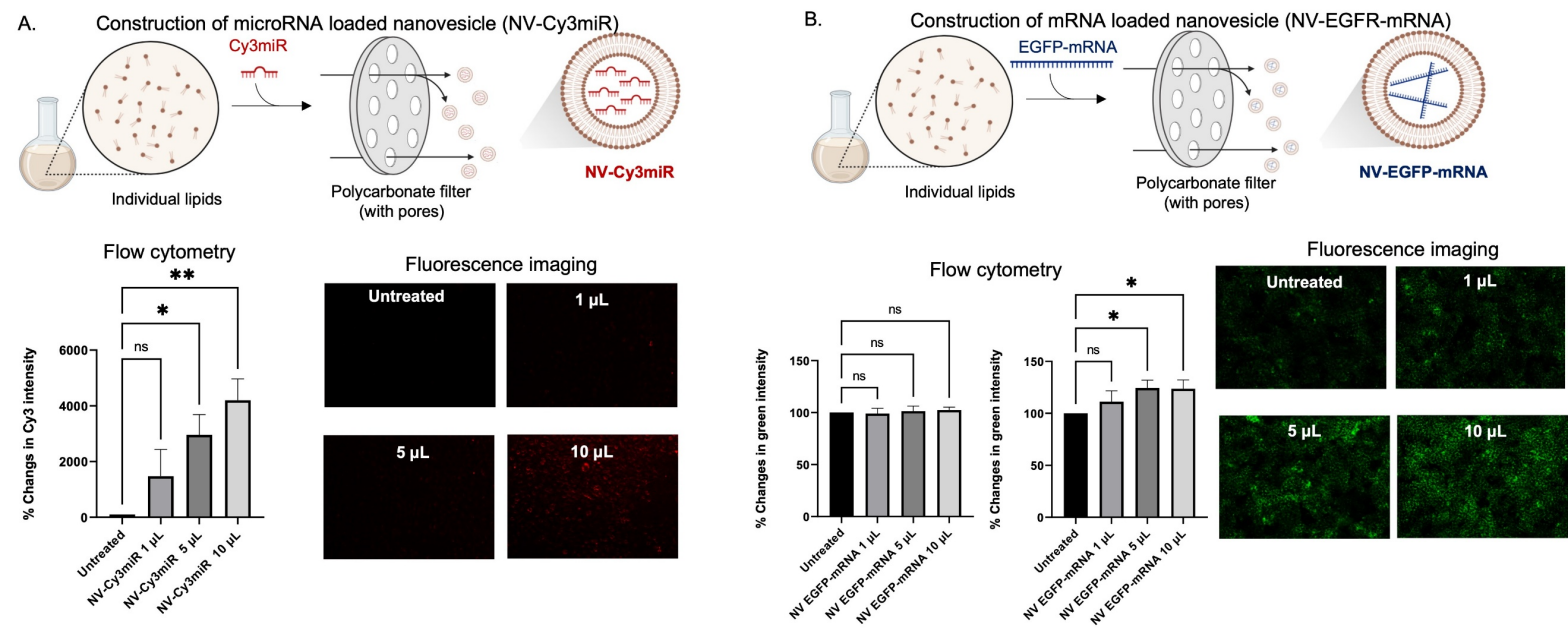
RNA encapsulations and delivery potentials of the nanovesicle. (A) Schematic illustration of the construction of microRNA (Cy3 labelled) loaded nanovesicles (NV-Cy3miR) along with flow cytometric analysis and fluorescence imaging of HaCaT cells being incubated with different amounts of NV-Cy3miR for 24 h. (B) Schematic illustration of the construction of mRNA loaded nanovesicles (NV-EGFP-mRNA) along with flow cytometric analysis of the HaCaT cells after being incubated with different amount of NV-EGFP-mRNA for 24 h and 48 h, and fluorescence imaging at 48 h of incubation. (ns = not significant; *p < 0.05, **p < 0.01 by un pair t-test).

As the efficiency of cellular uptake of the DNA stands had been illustrated (Figure 3), we became interested in investigating the functions to determine whether they can be implemented for delivering other nucleic acids, i.e., mRNA and microRNA. Like DNA loading, miRNA and microRNA loaded nanovesicles were also obtained by the extrusion method (as depicted in Figure 4A-B). By incubating the cells with different amounts of microRNA loaded nanovesicles (NV-Cy3miR) for 24 h, the fluorescent intensity was found to increase in a dose-dependent manner and was significantly increased at higher concentrations (Figure 4A, bottom panel). In contrast, some nucleic acids, especially mRNA, displayed unacceptable outcomes at 24 h (Figure 4B, bottom left panel). However, extending the duration of nanovesicle's incubation to 48 h revealed a statistically significant difference in green fluorescent intensity (Figure 4B, bottom right panel). Since most of nucleic acids were able to be delivered by the nanovesicles, some nucleic acids, i.e., mRNA might not connect with this sort of alignment since their size might be a limitation. Indeed, configuring multilayers and increasing the complexity of nanovesicles has been being attempted by existing studies since this strategy allows for superb functionality of drug delivering and for mRNA strands, in particular 4. Fortunately, our approach revealed acceptable outcomes towards mRNA loading and delivery to cell target regardless of size limitations of mRNA.

**Figure 5:**
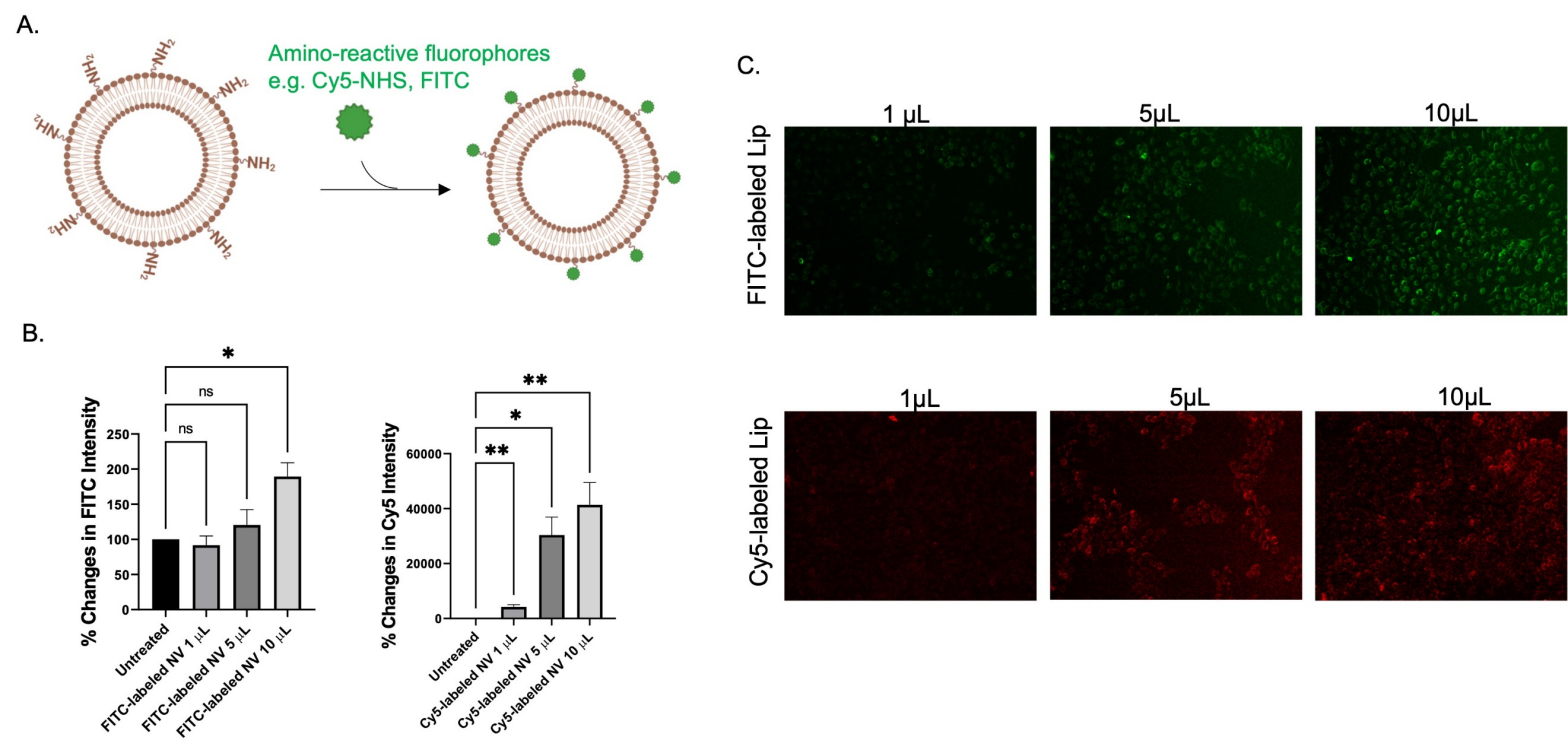
Bioconjugation capability of the nanovesicle. (A) Schematic illustration of the labeling of the nanovesicles with amino-reactive fluorophores such as FITC and Cy5-NHS ester. (B) flow cytometric analysis and (C) fluorescence imaging of the HaCaT cells after being incubated with different amount of FITC labeled nanovesicle and Cy5 labeled nanovesicle for 48 h. (ns = not significant; *p < 0.05, **p < 0.01 by unpair t-test).

To date, nanovesicles can be applicable for precision medicines as they provide targeted delivering properties. In this context, targeting nanovesicles seems to be applied to current situations as it provides greater efficiency of drug delivery systems. Interestingly, our previous FTIR results revealed that amine and amide were substantial on nanovesicle (Figure 1E). Subsequently, a study was completed on how to implement these functional groups. As amine nucleophilic parts play crucial roles in bioconjugation using suitable chemistry. Amino-reactive fluorophores such as FITC and Cy5-NHS ester were applied for labeling pure nanovesicles (Figure 5). Flow cytometric analysis and fluorescent imaging demonstrated that fluorescent intensity of the cells was found to increase as the amount of FITC- and Cy5- labeled nanovesicles increases (Figure 4B, 5C). According to the results, there were committed characteristics of nanovesicle conjugating ability based on different methods of conjugation and types of fluorescent marker used.

## Conclusion

In this work, we showed that nanovesicles can be successfully prepared by using breast milk as a raw material without the use of any synthetic substances. The production process of the nanovesicle was done without using any chemical solvents, starting from lipid isolation by repetitive freeze-thaw, centrifugation, and sonication, followed by an extrusion thru a polycarbonate membrane, and finally purification by a column filter. The milk derived nanovesicle exhibited a small in size with good size distribution, and a negative surface charge. Cellular response determinations revealed that milk-derived nanovesicles had no harmful effects on cell viability and ROS generation in HaCaT cells and on immune responses in PBMCs. The nanovesicles were capable of delivering different nucleic acids such as single strand DNA, microRNA, and mRNA into HaCaT cells. This outcome also consolidated the power of drug delivering by natural biomolecules that were able to perform cellular biocompatibility, were less immunogenic, and were simple to prepare, etc. Furthermore, nanovesicle's structures displayed great possibility for molecular interaction. These findings provided useful insight regarding the implementation of a chemically-modified approach toward prospective target molecules. Therefore, extensive applications of nanovesicles are likely to be profitable as a means of biomolecular control and especially for personalized medicine in the future.
